# Human cystatin C induces the disaggregation process of selected amyloid beta peptides: a structural and kinetic view

**DOI:** 10.1038/s41598-023-47514-w

**Published:** 2023-11-27

**Authors:** Adriana Żyła, Anne Martel, Przemysław Jurczak, Augustyn Moliński, Aneta Szymańska, Maciej Kozak

**Affiliations:** 1 Department of Biomedical Physics, Faculty of Physics, Adam Mickiewicz University, Poznań, Poland; 2 NanoBioMedical Centre, Adam Mickiewicz University, Poznań, Poland; 3 Large Scale Structures, ILL Neutrons for Society, Institute Laue-Langevin, Grenoble, France; 4 Laboratory of Medical Chemistry, Department of Biomedical Chemistry, Faculty of Chemistry, University of Gdańsk, Gdańsk, Poland; 5 SOLARIS National Synchrotron Radiation Centre, Jagiellonian University, Kraków, Poland

**Keywords:** Biophysics, Molecular biophysics, Biological physics

## Abstract

Neurodegenerative diseases, such as Alzheimer’s disease (AD) and various types of amyloidosis, are incurable; therefore, understanding the mechanisms of amyloid decomposition is crucial to develop an effective drug against them for future therapies. It has been reported that one out of three people over the age of 85 are suffering from dementia as a comorbidity to AD. Amyloid beta (Aβ), the hallmark of AD, transforms structurally from monomers into β-stranded aggregates (fibrils) via multiple oligomeric states. Astrocytes in the central nervous system secrete the human cystatin C protein (HCC) in response to various proteases and cytokines. The codeposition of Aβ and HCC in the brains of patients with AD led to the hypothesis that cystatin C is implicated in the disease process. In this study, we investigate the intermolecular interactions between different atomic structures of fibrils formed by Aβ peptides and HCC to understand the pathological aggregation of these polypeptides into neurotoxic oligomers and then amyloid plaques. To characterize the interactions between Aβ and HCC, we used a complementary approach based on the combination of small-angle neutron scattering analysis, atomic force microscopy and computational modelling, allowing the exploration of the structures of multicomponent protein complexes. We report here an optimized protocol to study that interaction. The results show a dependency of the sequence length of the Aβ peptide on the ability of the associated HCC to disaggregate it.

## Introduction

The development of neurodegenerative disorders (e.g., Alzheimer's and Parkinson's diseases) correlates with the extended lifetime of our population. The current progress in medicine only slightly affects the long-term survival of patients diagnosed with Alzheimer’s disease. It is therefore of great importance to seek molecular level mechanisms that can contribute to the design of effective drugs or new therapeutic strategies.

Amyloid beta (Aβ) peptide deposits are the main component of senile plaques, commonly described as the histological fingerprint of Alzheimer’s disease (AD)^1^, Down’s syndrome^2^, or type II diabetes^3^. Their occurrence in the brains of patients during the development of Alzheimer's disease provided the basis for creation of "the amyloid hypothesis" that associated their presence with the pathogenesis of Alzheimer's disease^4^. However, the long-term search for potential therapeutic methods aimed at removing amyloid plaques was not successful, which focused the researchers' interest on the neurotoxicity of soluble Aβ a peptide oligomers^5,6^. Aβ deposits disrupt neuronal cell homeostasis and the transmembrane flow of ions^7^. The presence of soluble toxic amyloid oligomers and deposits initiates the process of apoptosis in neuronal cells, causing their death and leading to dementia^8^. It is known that the pathological variant of the Aβ peptide appears in the extracellular space as a result of the amyloidogenic degradation pathway (proteolytic cleavage by β-secretase and **γ**-secretase)^9^ of amyloid precursor protein (APP). The main components of the senile plaques include Aβ 1–40, Aβ 1–38, and Aβ 1–42 fragments. Other, less abundant variants are Aβ 1–37 and Aβ 1–43^10^. Different oligomeric states of Aβ peptide (polymorphic oligomers, protofibrils, and fibrils) are present in the intracellular space. Moreover, this structural diversity endows it with a range of properties, including different lifetimes and neuronal toxicity levels. Recently, the amyloid hypothesis was discussed again by Karran and De Strpper^11^, who emphasized that Aβ pathology drives Tau pathology, so amyloid plaque should be reduced to a low level, which is important in the context of new, more effective therapies.

The polymorphism of Aβ fibrils depends on the amino acid sequence of peptides involved in the formation of the “steric zipper” and the “cross-β spine” within the fibril structure^12^. All amyloidogenic polypeptides can form the same fibril core with a similar molecular arrangement^13^. It is now commonly known that misfolded oligomers (known as "seeds") or, in particular, Aβ peptides in beta-sheet oligomeric form, lead to a chain nucleation reaction similar to prion peptide infection^14^. There is also some evidence that Aβ oligomers can induce a similar reaction to Tau protein misfolding^15^, a biological marker of Parkinson’s disease^16^.

Breaking down amyloid plaques and reducing the formation of Aβ oligomers is a potential way to slow or inhibit the progression of Alzheimer's disease. Therefore, factors capable of breaking down amyloid structures have been intensively researched. Recent studies revealed that Aβ in amyloid plaques in the brain tissue and the cerebrospinal fluid (CSF) of AD patients is accompanied by other proteins, including also human cystatin C (HCC). This small globular protein (MW = 13.3 kDa), secreted by all nucleated cells and present in all body fluids, including the CSF, is involved in controlling the enzymatic activity of cysteine proteases^17^. Intense neuronal HCC immunoreactivity has been observed in cortical neurons of AD patients, particularly in regions most severely affected by the disease, such as the temporal and entorhinal cortical areas and the hippocampal region^18^. In addition, these conglomerates can be observed, in some cases, even in healthy subjects^19^. The HCC concentration in CSF is elevated in homeostatic conditions, which might suggest an important protective role in the central nervous system^20^. Moreover, it has been observed that HCC promotes neural stem‐cell growth. Typically, the concentration of HCC in the CSF of healthy adults oscillates at approximately 5.8 mg/L, which is 5–6 times higher than that in plasma^21,22^. Increased HCC concentrations in CSF have been reported in patients with infectious nervous disorders^23^. The pathogenic form of HCC is manifested by the Leu68Gln mutation, where a hydrophobic amino acid residue is replaced by polar and bulkier one in the hydrophobic core of HCC, which results in development of serious disease—hereditary cystatin C amyloid angiopathy (HCCAA)^24^.

**Figure 1:**
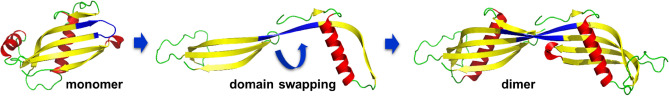
Schematic representation of the domain swapping mechanism—the monomeric form of human cystatin C (V57G mutant; PDB code: 6ROA^40^) is converted into dimeric forms of protein (native HCC; PDB code: 1TIJ)^28^. The flexible residues involved in domain swapping are marked in navy blue.

The structure of human cystatin C is flexible and very susceptible to conformational changes and oligomerization through a domain-swapping mechanism (see Fig. 1), forming dimers, trimers, oligomers and fibrils^25^. The monomeric state of human cystatin C is a native, functional state of this protein in body fluids^25^. HCC only in the monomeric form is fully effective as an inhibitor of proteases, however, significant amounts of extracellular HCC dimers are present in pathological conditions^26^.

The tree-dimensional structures of several molecular variants of HCC, were characterized by X-ray crystallography and NMR spectroscopy^27^. First obtained structure of the wild type HCC (wt HCC) was a domain-swapped dimer which crystallized in two polymorphic crystal forms^25,28,29^. The global conformation of the wt HCC dimer is diverse—the tetragonal form^28^ forms an extended dimer and the structure of more compact HCC dimer is observed for cubic polymorph^25^. The structure of HCC dimers with 3D domain swapping was also observed for several point mutants of (Val57Asp andVal57Pro)^30^ as well as for N-truncated HCC form^31^. Unfortunately, the spatial structure of the wt HCC monomer was not characterised so far, however, monomeric structures have been obtained for point mutants in the hinge loop area, which protect molecule against domain swapping (Val57Gly^27^, Val57Asn^32^) and also for the monomeric form of HCC *stab1* variant with an additional engineered disulfide bond introduced (Leu47Cys)-(Gly69Cys)^33^. Domain swapping is however probably not a critical parameter for cystatin to undergo the oligomerisation. Results obtained by Pearlfein et al*.*^34^ for stabilised against domain swapping HCC mutant (Val57Asn) showed the possibility of the formation of oligomeric forms, however without domain swapping, since the oligomers retained the inhibitory activity.

Conformational changes leading to HCC oligomerization are characteristic of the disease state (e.g. as for Leu68Gln variant in HCCAA)^35^ but can also be induced in vitro via X-ray irradiation^36^. Higher oligomers (trimers, decamers, dodecamers) were observed and characterized at the microscopic level^37–39^. The oligomerization and formation of fibril-like structures of HCC are strongly related to its β-sheet secondary structure and hydrophobic interactions occurring within the protein^28^.

The correlation between Aβ and HCC was supported by the discovery of the direct binding of the proteins into a 1:1 molar high‐affinity complex^41^. Kaeser and co-workers^42^ communicated that HCC overexpression in brains of APP-transgenic mice reduced deposition of cerebral amyloid-β and HCC is able to bind amyloid-β and inhibit Aβ fibril formation. Also Mi et al.^43^ presented data that HCC binds soluble amyloid-β peptide and inhibits formation of cerebral amyloid in APP-transgenic mice. In a later study performed by Tizon et al.^44^, it was indicated that the extracellular addition of HCC in presence of preformed oligomeric or fibrillar Aβ forms increased cell survival. They underlined that HCC inhibits Aβ aggregation, however HCC is not able to dissolve preformed Aβ fibrils or oligomers.^44^ It is also worth to note interesting observation communicated by Wang and co-workers^45^, that HCC reduced Aβ40 secretion in human brain microvascular endothelial cells. However, in this case HCC induced reduction of Aβ40 level was caused by degradation of β-secretase activity (BACE1).

Despite these interesting from a point of view of potential anti-amyloid therapeutics observations, the mechanism of Aβ-HCC direct and indirect interactions at molecular level as well as the structure of Aβ-HCC complex have not yet been thoroughly fully characterized.

At this point, it should be considered whether human cystatin C is able to block the formation or break down of existing amyloid Aβ fibrils. The aforementioned presence of HCC in amyloid deposits could potentially indicate such an effect. Therefore, the aim of our study was to visualize interactions in solution between HCC and fibrils formed by selected amyloid beta peptides using a combination of small-angle neutron scattering (SANS), atomic force microscopy (AFM) and modelling. For our study we selected two Aβ peptides, Aβ 1–42, the most abundant peptide variant found in amyloid deposits and Aβ 3–28 peptide, also forming stable fibrils. This second short synthetic peptide contains central hydrophobic region (residues 16–22) important in oligomerisation process and lysine K28^46,47^. The results showed a dependency of the sequence length of the Aβ peptide creating the fibril and the ability of the associated HCC to depolymerize them. We discovered that HCC may be a guardian of the CNS and not only inhibit amyloidogenic peptide aggregation but also depolymerize some deposits what could provide protection against further toxic influences on neuronal cells. We believe that understanding the mechanisms of the complexation of human cystatin C with Aβ will provide more insight into possible ways to stop or delay this pathogenic process.

## Materials and methods

### Synthesis of Aβ peptides

All peptides were synthesized by means of solid-state synthesis using an automatic, microwave-assisted peptide synthesizer (Liberty Blue, CEM) and purified with RP-HPLC. An XBridge Prep C8, 5 µm OBD (Waters) column was used, and peptides were eluted with an appropriate gradient of buffers A and B, where buffer A is composed of 5 mM NH_4_HCO_3_ in water with pH 10.5 (adjusted with ammonium hydroxide) and buffer B is 80% acetonitrile in buffer A. The column was operated by a Shimadzu chromatography system (LC-20A pump, SPD-20A detector and CBM-20A communication bus module). After purification, the obtained peptides were lyophilized, and their level of purity was verified with analytical chromatography using an XTerra RP_8_ column (5 µm, 4.6 × 250 mm, Waters) operated by a Nexera-i chromatography system (Shimadzu). We were able to produce high-quality peptides with a purity above 96%.

### Preparation of Aβ samples

#### Preparation of monomeric form of Aβ peptide

To trigger Aβ peptide disaggregation into its monomeric form, hexafluoro-2-propanol (HFIP) was used. HFIP is a strong acid that effectively disrupts the secondary structure of aggregated peptides and causes them to unfold; therefore, we used a modification of a previously published protocol of Aβ disaggregation^48^. First, a solution containing 1 mg of Aβ 1–42 or Aβ 3–28 peptide per 1 mL of ice-cold HFIP (Sigma Aldrich) was prepared and sonicated twice for 15 min in a bath sonicator (Ultrasonic Bath Elmasonic) on ice. After the sonication step, the peptide/HFIP solution was divided into small aliquots and evaporated, and vials with dry peptide films were stored at − 20 °C for future experiments. For experiments presented here, these fractions were freshly prepared and stored no longer than the 5 days before using.

#### Aβ peptide aggregation

The fibrillary Aβ 1–42 or Aβ 3–28 peptides were the main objects of our study; therefore, a standard fibrillation protocol was applied^49^. To obtain fibrils of Aβ peptides, a solution of the monomeric form of Aβ peptide (1 mg/mL) was incubated for up to 48 h at 40 °C under gentle agitation. The oligomeric (fibrillary) state of Aβ 1–42 and Aβ 3–28 peptides was assessed using atomic force microscopy. The aggregated peptide samples were then used for SANS kinetics studies and imaging by atomic force microscopy (AFM).

### Production and purification of ^2^H-labelled HCC (D-HCC)

Initially, the standard HCC expression and purification protocol was tested^50^. The DNA containing the HCC wild-type gene, ampicillin resistance gene, and temperature promoter was transformed and expressed in *Escherichia coli* BL21 (DE3) competent cells (Novagen; Sigma Aldrich). For the expression of ^2^H-labelled protein, a modified protocol described by Marley et al.^51^ was used. As described in a previous publication^40^, transformed *E. coli* culture was incubated at 32 °C until the OD_600_ = 0.4. Next, the bacterial suspension was transferred into M9 minimal medium^52^ prepared with heavy water (D_2_O) as a solvent and cultured until the OD = 0.6. Then, the incubation was continued at 42 °C for another 3 h. The isolation and purification of the expressed HCC wild-type proteins were performed with a two-step procedure involving the use of ion-exchange chromatography and size exclusion chromatography^53^.

The protein produced with the described procedure exhibited a high level of deuteration and purity; however, the protocol was not particularly efficient due to the incompatibility of ampicillin with D_2_O-based media. Ampicillin resistance plasmids are not recommended in high cell density culture protocols with deuterated media because the secreted beta-lactamase degrades antibiotics and causes overgrowth of plasmid-free cells^54^. Therefore, another approach was undertaken. To optimize protein production, we successfully applied the “high-yield expression” procedure proposed by Cai et al.^55^, where HCC overexpression occurred at a high bacterial growth level (high OD_600_ value) and at a low culture volume. The newly designed and more robust protocol for HCC production was applied. The pET-24(a) plasmid containing the HCC gene, kanamycin resistance gene, and IPTG-controlled expression promoter was transformed into Shuffle T7B (New England Biolabs) *E. coli* cells—a strain with enhanced capacity to correctly fold proteins with multiple disulfide bonds (e.g., HCC) within its cytoplasm. To mitigate the extreme sensitivity of the Shuffle strain to the level of oxygen dissolved in D_2_O media, different media were tested, and TB medium was selected. The transformed bacterial cells were cultured on LB agar plates at 18 °C for 48 h. Next, a single colony was selected and transferred to 25 mL of LB medium (H_2_O) containing 50 mg/mL kanamycin and cultured overnight at 30 °C with agitation. The medium was then centrifuged at low rpm (4668 g), and the cell pellet was resuspended in fresh TB medium containing 80% D_2_O and 20% deionized water (v/v) as a solvent with 100 mg/L kanamycin. The cell culture was then incubated at 30 °C with shaking (220 rpm). The OD_600_ was measured every 2 h, and induction using 0.2 mM IPTG was performed when the culture optical density reached OD = 5–6. Then, the cells were incubated at 18 °C and harvested after another 20 h. The bacterial pellet was resuspended in lysis buffer (20 mM Tris–HCl, pH 7.5) and sonicated (Sonoplus ultrasonic homogenizer, Bandelin) for 5 min (pulse 3 s with 2 s break, ultrasound intensity 20% of amplitude) in an ice bath. Next, the suspension was centrifuged for 30 min at 14000 rpm, and the crude lysate was purified with ion-exchange chromatography with HiTrap Q (the flow-through was collected). The purified fractions were then dialyzed against 20 mM ammonium bicarbonate buffer (pH 7.8) and lyophilized. The lyophilized protein was then again diluted in ammonium bicarbonate buffer and purified with size exclusion chromatography with a Superdex 75 10/300 GL column (Cytiva). The presence of D-HCC protein in all the fractions obtained after each purification step was monitored using SDS–PAGE electrophoresis.

### Small-angle neutron scattering data collection

**Figure 2:**
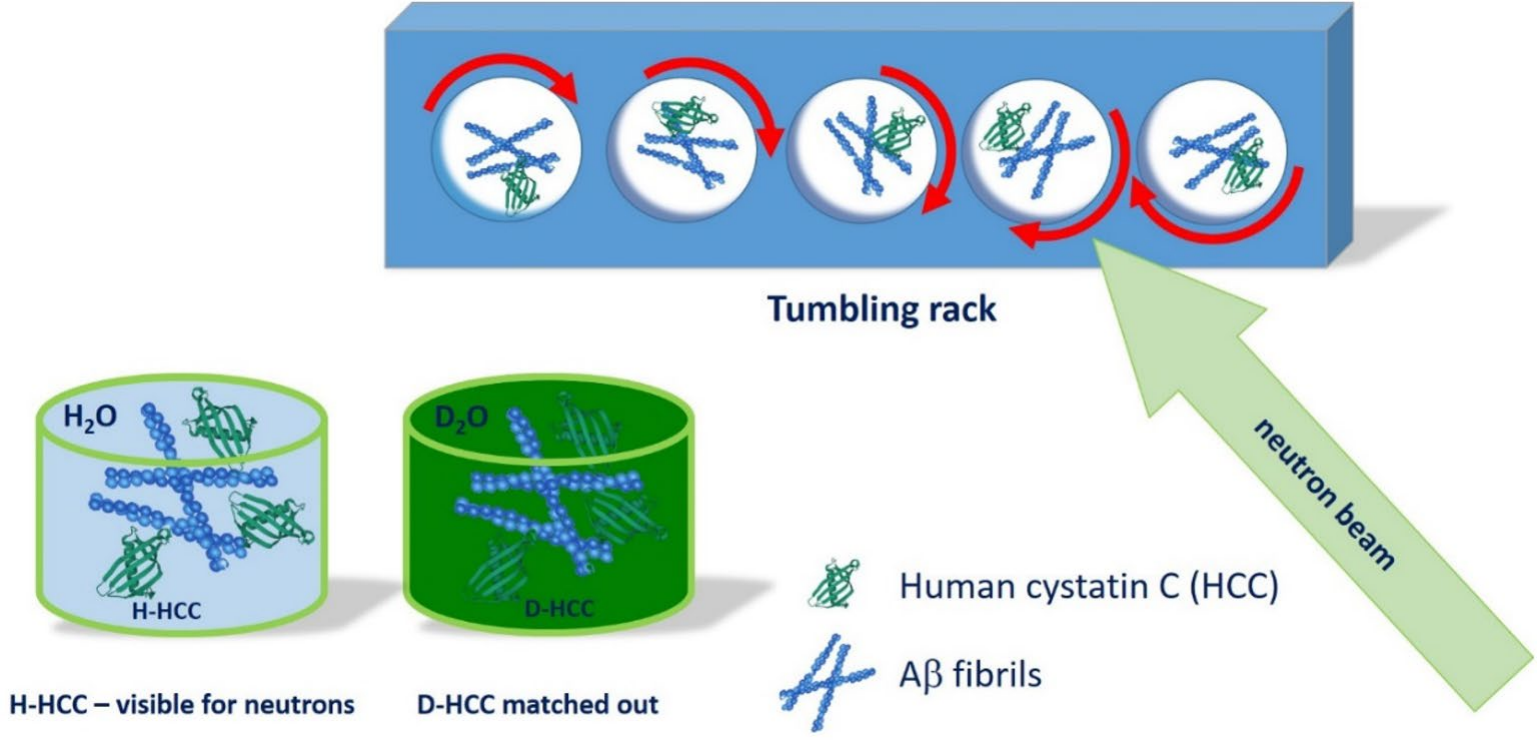
Schematic representation of the SANS experimental setup. The tumbling rack was applied to prevent peptide fibril sedimentation. For samples containing D-HCC, only SANS profiles of Aβ fibrils in match point conditions were observed.

The collection of solution SANS data for Aβ 1–42 or Aβ 3–28 peptides and their mixtures with HCC (protonated (^1^H)- or deuterium (^2^H)-labelled HCC) were performed on the D22 beamline at the Institute Laue-Langevin (ILL), Grenoble (France). Approximately 400 µL of sample was used to fill 1 mm thick banjo-type quartz cuvettes (Hellma 120-000-1-40), which were then closed without introducing any air bubbles. Cuvettes were placed in a tumbling rack to avoid fibril precipitation through the measurement (Fig. 2). Neutron scattering was collected at a wavelength of 6 Å ± 10% and two sample-detector distances (17.6 m and 5.6 m), with a symmetric collimation with a 40 mm by 55 mm cross section and a sample aperture of 13 mm diameter. In all configurations, a second detector was present 1.4 m from the sample to register large q data.

### SANS kinetics and static measurements

To prepare fibrils, monomeric Aβ peptide was diluted in water to a concentration of 0.1 mg/mL and incubated under agitation for 48 h at 40 °C. The fibrils were lyophilized and resuspended in D_2_O buffer to an estimated Aβ concentration of 1 mg/mL. The sample with a suspension of fibrils only and a mixture of fibrils with H-HCC or D-HCC in 100% D_2_O PBS buffer was dispensed in quartz cuvettes and placed into a tumbling rack. The rotation of the sample during measurement avoids sample sedimentation during a long exposure time (Fig. 2). The SANS kinetics measurements were performed at 37 °C, and data were collected for Aβ fibrils (Aβ 1–42 and Aβ 3–28) only and fibrils in the presence of deuterated HCC (at a concentration of 1 mg/mL). Each scattering profile in kinetics measurements was recorded for 12 min. The static measurements of fibrils in the presence of H-HCC and D-HCC (1.5 mg/mL) were performed at room temperature. Each scattering profile in static measurements was recorded for 12 min.

The analysis of the collected data was performed with the use of GRASP,^56^ Igor Pro 8 software with macros created for SANS data analysis^57^ and SasView from the ATSAS package^58^. Further analysis and interpretation of the SANS data was carried out by fitting the scattering intensity of the SANS data (I) in double logarithmic plot to power law (I ≈ q^−α^), where q is a scattering vector and α is a scattering exponents^59^. Especially the relation between α and specific types of structures (fractal, aggregates, etc.) was analysed, similarly as was used previously in analysis of other fibrillar or polymeric systems^60–62^.

### Modelling and visualization of Aβ fibrils

To visualize and compare the experimental results with the theoretical data, we chose a model of Aβ fibrils (Aβ 1–40 PDB code: 6SHS^63^). The model strongly correlates with the collected experimental data. To visualize the Aβ 3–28 fibril, we adjusted the sequence of the Aβ 1–40 PDB structure and performed molecular dynamics (MD) simulations with Unress^64^ to optimize the new structure. Unress is a coarse-grain method allowing rapid MD simulation for relatively large molecular complexes. To perform the molecular docking of monomeric Aβ peptides to the HCC structure, we used CabsDock software^65^, allowing the docking of a flexible amino acid chain to the receptor–protein 3D structure. The restraints of the interaction between Aβ and HCC reported by NMR studies^66^ were enforced in the simulation. The molecular structures were visualised using PyMOL Molecular Graphics System (Schrödinger, LLC).

### Atomic force microscopy

The fibrillary samples of Aβ 1–42 or Aβ 3–28 peptides and their mixtures with human cystatin C in a molar ratio of 1:1 were diluted (1:1000, where initial sample concentrations were ~ 1 mg/mL) in deionized water, and then these solutions were deposited (sample volume approximately 10 µL) onto a freshly prepared mica surface (mica V1 grade, Ted Pella INC.). The samples deposited on mica were dried for a few hours under the cover at room temperature. For AFM imaging, we applied a modified procedure, used previously by us for imaging of human cystatin C oligomers^37,38^. All experiments were carried out with a Nanowizard IV atomic force microscope (JPK, Berlin, Germany) optimized for biological imaging. Aβ peptide fibrils and their mixtures with human cystatin C were studied using a Tap150-G silicon soft tapping mode AFM probe (BudgetSensors Innovative Solutions Bulgaria Ltd.) and the intermittent (air) contact mode. The visualization and analysis of the topographic images of the samples were carried out using Gwyddion 2.60 modular software for SPM data analysis^67^.

## Results

In our study, we used a combination of experimental methods and molecular modelling to describe the influence of HCC on Aβ 1–42 and Aβ 3–28 peptide fibrils, present the morphology of the complexes formed by selected Aβ peptides and HCC as a result of their interactions and visualize the Aβ peptide fibril disaggregation process induced by HCC.

Initially, an effective protocol for high-yield expression of deuterated human cystatin C in *E. coli* (Shuffle T7B strain) was implemented. This allowed us to produce approximately 5 mg of purified D-HCC from 400 millilitres of culture. Detailed results of the production and purification of D-HCC are summarized in the Supplementary data (see Figs. S1 and S2).

### Initial models of Aβ fibrils

**Figure 3:**
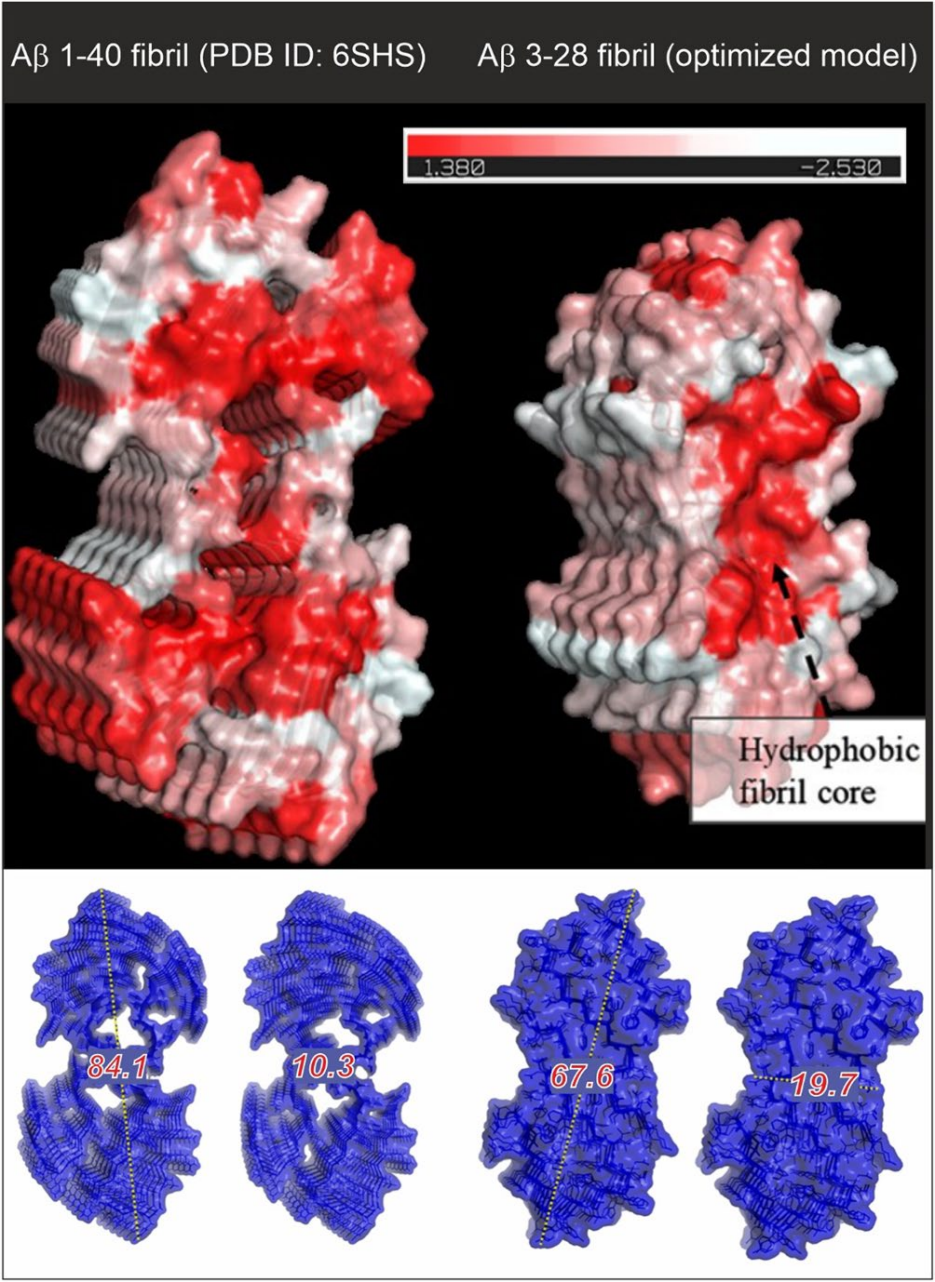
Cross section with the hydrophobic representation of Aβ fibrils. The hydrophobic residues are coloured in red (see hydrophobicity scale). On the left (down)—the experimental model from the cryo-TEM^46^, on the right (down)—the structure obtained by homology modelling and MD structure optimization (this work). The figure was created using PyMOL Molecular Graphics System, (Schrödinger, LLC).

Currently, there are only a few spatial structures of Aβ peptides registered with NMR spectroscopy methods (PDB deposits: 6SZF^68^, 2MXU^69^, 2NAO^70^, 6Y1A^71^), as well as only one structure of Aβ amyloid fibrils purified from Alzheimer's brain tissue obtained using cryo-electron microscopy (PDB: 6SHS^63^). Amyloid fibrils can also be easily detected and characterized with atomic force microscopy^72^. However, amyloid peptides are very hard to characterize due to the nucleation reaction, which can start very rapidly. Moreover, the difficulties in the description of structural mechanisms of Aβ aggregation result from the complexity of fibril polymorphism. Registering the structure of the complexes formed between Aβ fibrils and other biomolecules with high-resolution methods (e.g. protein crystallography) is close to impossible because of the high level of sample polydispersity. Therefore, for the structural characteristics of Aβ fibrils, the cryoEM model of Aβ 1–40 fibrils was used. The atomic model of the Aβ 3–28 structure was obtained by a homology modelling approach using Aβ 1–40 as a template. The model was optimized using molecular dynamics (MD) simulation by the Unress^64^ method. Both these structures and their structural parameters (dimensions along the vertical and horizontal axes of the cross section) are presented in Fig. 3.

### SANS

Human cystatin C is susceptible to radiation damage induced by synchrotron radiation in standard SAXS experiments^36^. SANS is a powerful method that allows the study of structures of complexes formed between biomolecules in solution with the application of contrast variation and deuterium labelling^73^. In general, SANS and small-angle X-ray scattering are based on similar principles. However, SANS does not use a high-intensity X-ray radiation beam, which is a great advantage when studying radiation-sensitive macromolecules such as HCC protein. Additionally, without the use of radiation, SANS allows long kinetics experiments even at higher temperatures. We have developed a procedure with optimal parameters and environments to analyse the possible interaction between Aβ peptides and HCC.

**Figure 4:**
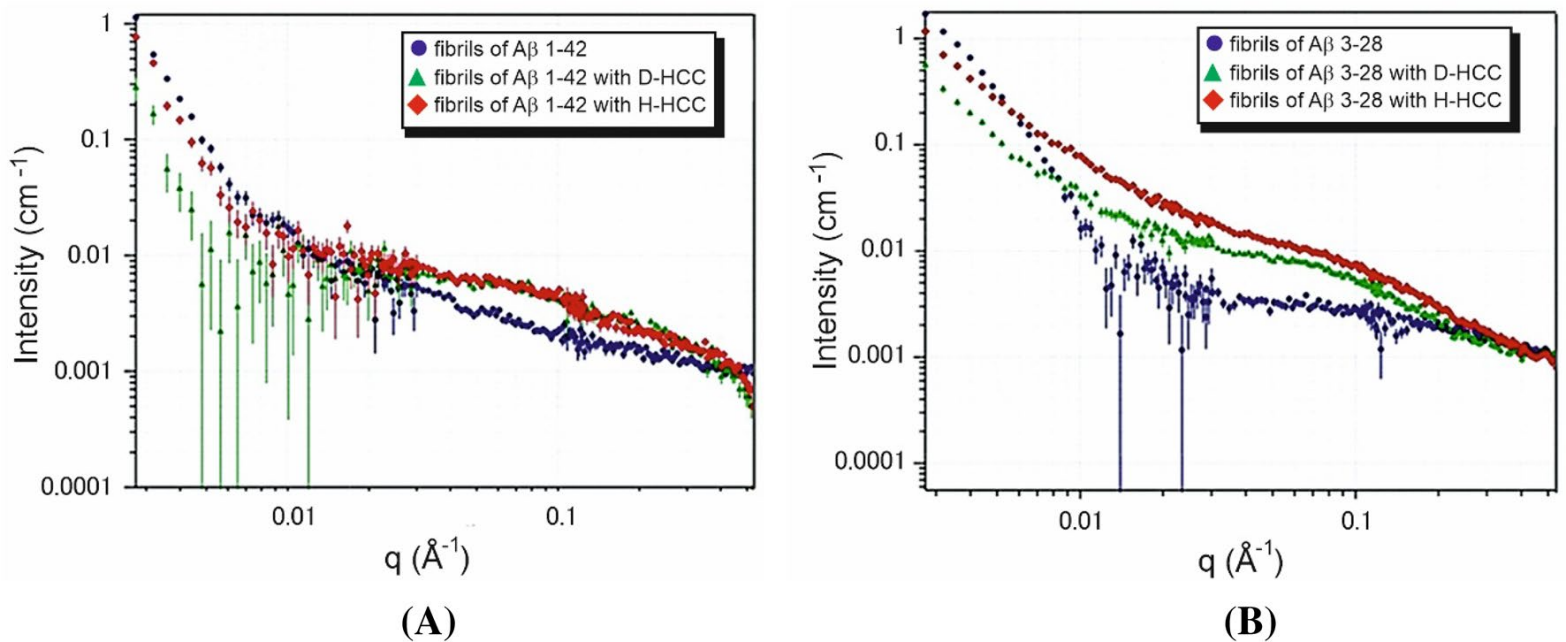
Comparison of SANS static experimental curves for Aβ fibrils alone and fibrils in the presence of H-HCC and D-HCC. (**A**) Aβ 1–42; (**B**) Aβ 3–28 fibrils.

**Figure 5:**
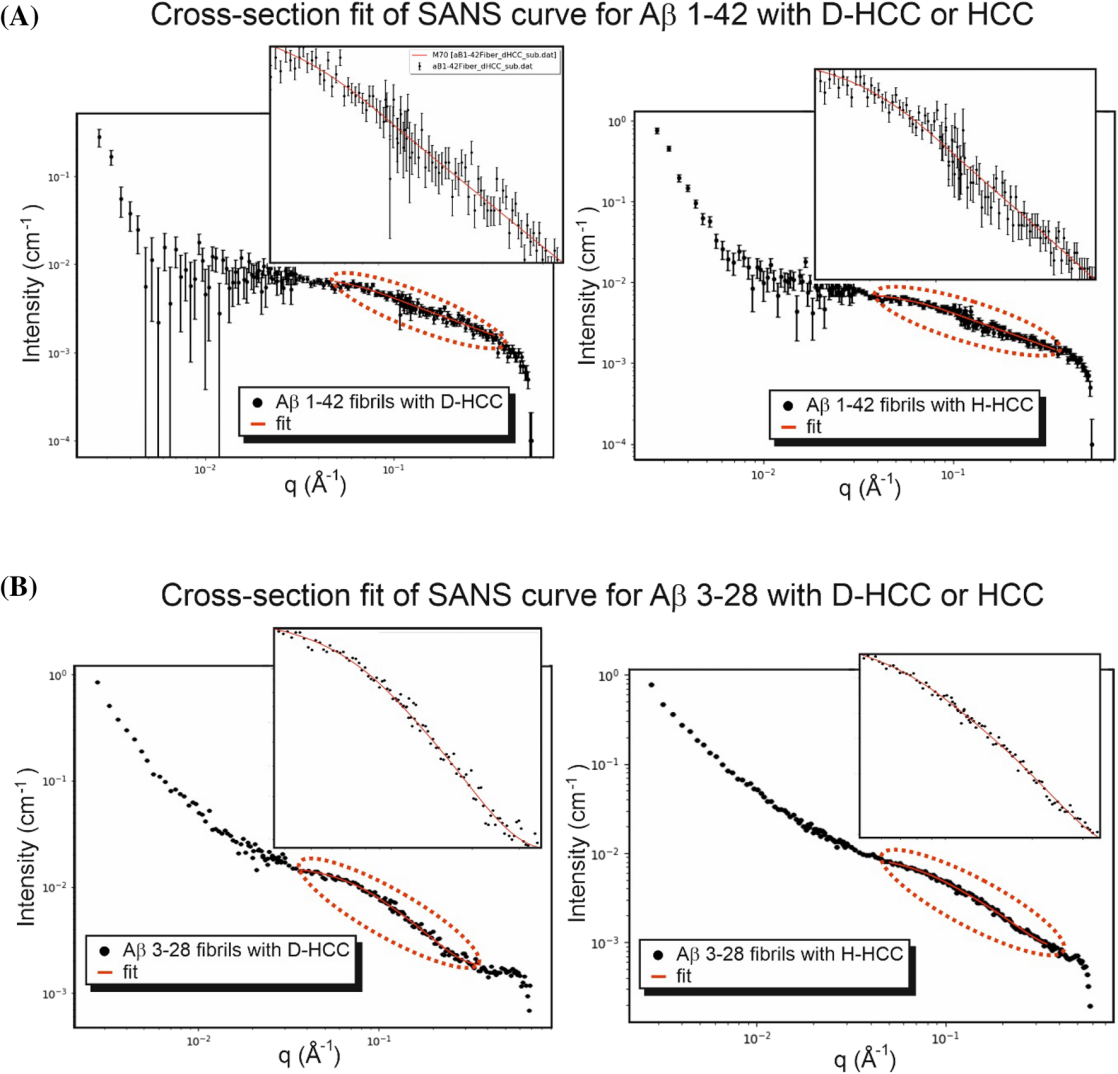
The analysis of small-angle neutron scattering data. Experimental SANS curves (blue) with the line of fit calculated from the ellipsoidal cylinder model (orange line) for experimental data of fibrils in the presence of H-HCC and D-HCC. (**A**) Aβ 1–42; (**B**) Aβ 3–28 fibrils. Insets present zoom of fitted regions of scattering curves (dashed line).

Scattering curves obtained from SANS measurements analysed according to power law show a nearly linear change with a slope of ~ 4 for Aβ 1–42 and ~ 3 for Aβ 3–28 fibrils (Fig. 4). These slope values of the scattering curve are characteristic for objects with sharp interfaces, here well-defined and stiff fibrils (Aβ 1–42) or 3D fractals (Aβ 3–28) such as probably a 3D network formed by fibrils (Figs. S7 and S8 from Supplementary materials). Regarding the samples containing a mixture of fibrils and HCC protein, the slopes for Aβ 3–28 fibrils with HCC exhibit significantly lower values (α ~ 2). Such a change indicates that in the presence of cystatin C, Aβ 3–28 fibers disintegrate and form volume fractals, i.e. probably aggregates formed on the basis of disintegrating Aβ 3–28 fibrils. However, in the data collected for the samples of D-HCC and Aβ 1–42 fibrils, we observed a loss of signal, making the data difficult to interpret (Fig. 4). We suspect that D-HCC interacts or probably sticks to Aβ 1–42 fibrils, which makes fibrils match-out for neutrons. The fibrils interacting with HCC were analysed by AFM imaging (see AFM section). According to the principle of SANS and fitting the ellipsoidal cylinder (fibril) function, the scattering curve data at q ~ 0.1 provide information about the cross-section of particles (fibrils) in the solution^74^. Taking in account the collected AFM data together with SANS results we can suppose that HCC interact with protofibrils or fibrils bundles and separates them from larger, denser assembles thus allowing the observation of the cross-section of fibrils in the scattering profile. All published models showed that the cross-section of Aβ fibrils takes an elliptical shape. The data collected from the cross-section of the Aβ fibril with cryo-TEM (PDB ID: 6SHS)^63^ corresponds well with a cross-section function of the ellipsoid model fitted for the experimental scattering profile (Figs. 3 and 5). The Aβ 1–42 fibril is characterized by a polar radius of ~ 39 Å and equatorial radius of ~ 5 Å (Fig. 3, Table 1), which indicates a very thin groove in the helical-like fibril structure, visible in the proposed cryo-TEM model of fibrils created by the Aβ 1–40 variant (Fig. 3). The Aβ 3–28 fibrils seem to have a more collapsed structure, which is visible in the parameters of the fitted cross-section function as well as the one provided by the molecular dynamic model (Fig. 3, Table 1).

**Table 1:** Summary of parameters of Aβ fibrils models.

	Fitted Aβ fibrils + D − HCC [Å]	Fitted Aβ fibrils + H − HCC [Å]	CryoEM model [Å]
*1–42*			
Polar radius	39 ± 4	38 ± 4	42.1
Equatorial radius	4.8 ± 0.3	4.7 ± 0.3	5.1
χ^2^	0.95	0.84	
*3–28*			
Polar radius	38 ± 2	36 ± 2	33.8
Equatorial radius	10.4 ± 0.2	9.3 ± 0.2	9.9
χ^2^	0.93	0.86	

### Disaggregation process—kinetic study

**Figure 6:**
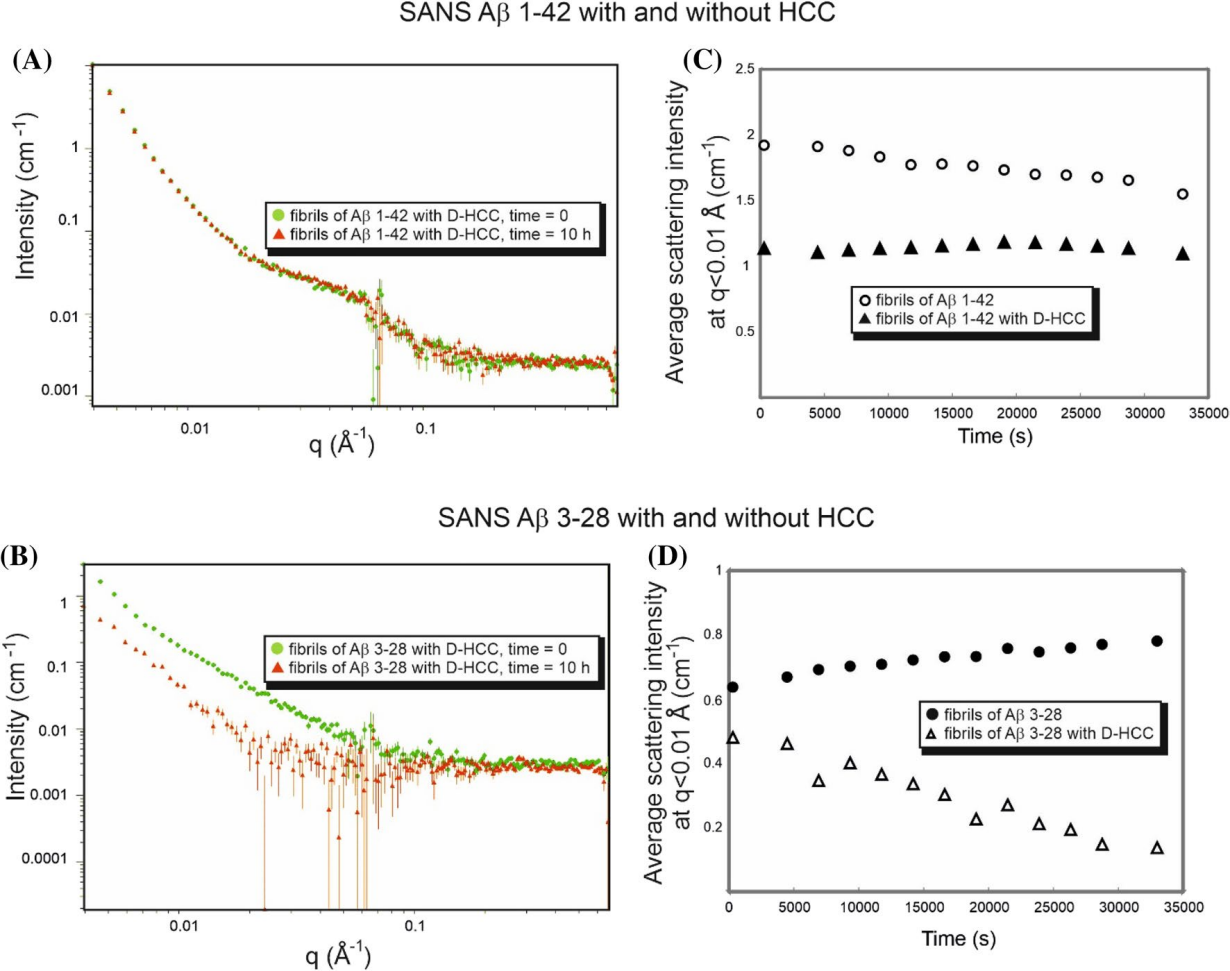
Plots showing data from the kinetics experiments for Aβ 1–42 and Aβ 3–28 fibrils with and without the presence of HCC. Plots (**A**) and (**B**) show a SANS profile collected at the beginning (green) and end (red) of the kinetics; plots (**C**) and (**D**) show a comparison of average intensity at q < 0.1 for each step of kinetics in time.

The data obtained from kinetic SANS experiments show a decreased intensity of signals for samples containing the mixture of fibrillar Aβ 3–28 and HCC. However, no decrease in signal intensity was observed for the reference samples Aβ 3–28 and Aβ 1–42 without HCC (Fig. 6A,B). Moreover, the sample containing a mixture of Aβ 1–42 and HCC also did not show any changes in signal intensity. Therefore, to visualize the kinetics of the disaggregation process, we calculated an average scattering intensity around q < 0.1 (5 data points) for each kinetics step. The average intensity values for each kinetic point were set together in a time resolution plot (Fig. 6C,D). The gradual decrease in the scattering intensity visible for the mixture of Aβ 3–28 with D-HCC during the time scale of 1–10 h indicates the gradual destruction (disaggregation) of the Aβ fibril structure. Analysing in details these changes in the scattering intensity at low values of the scattering vector (data at q < 0.01) in the SANS kinetic experiment, at the beginning (300 s) and at the end of the experiment, we observed over 70% decrease in the scattering intensity for the tested sample of Aβ 3–28 with HCC. However, for the Aβ 1–42 sample with HCC, we observe only a 4% decrease in the scattering intensity, which may indicate that the content of large particles (probably fibrils) practically did not change.

### Fibril disaggregation observed by AFM

**Figure 7:**
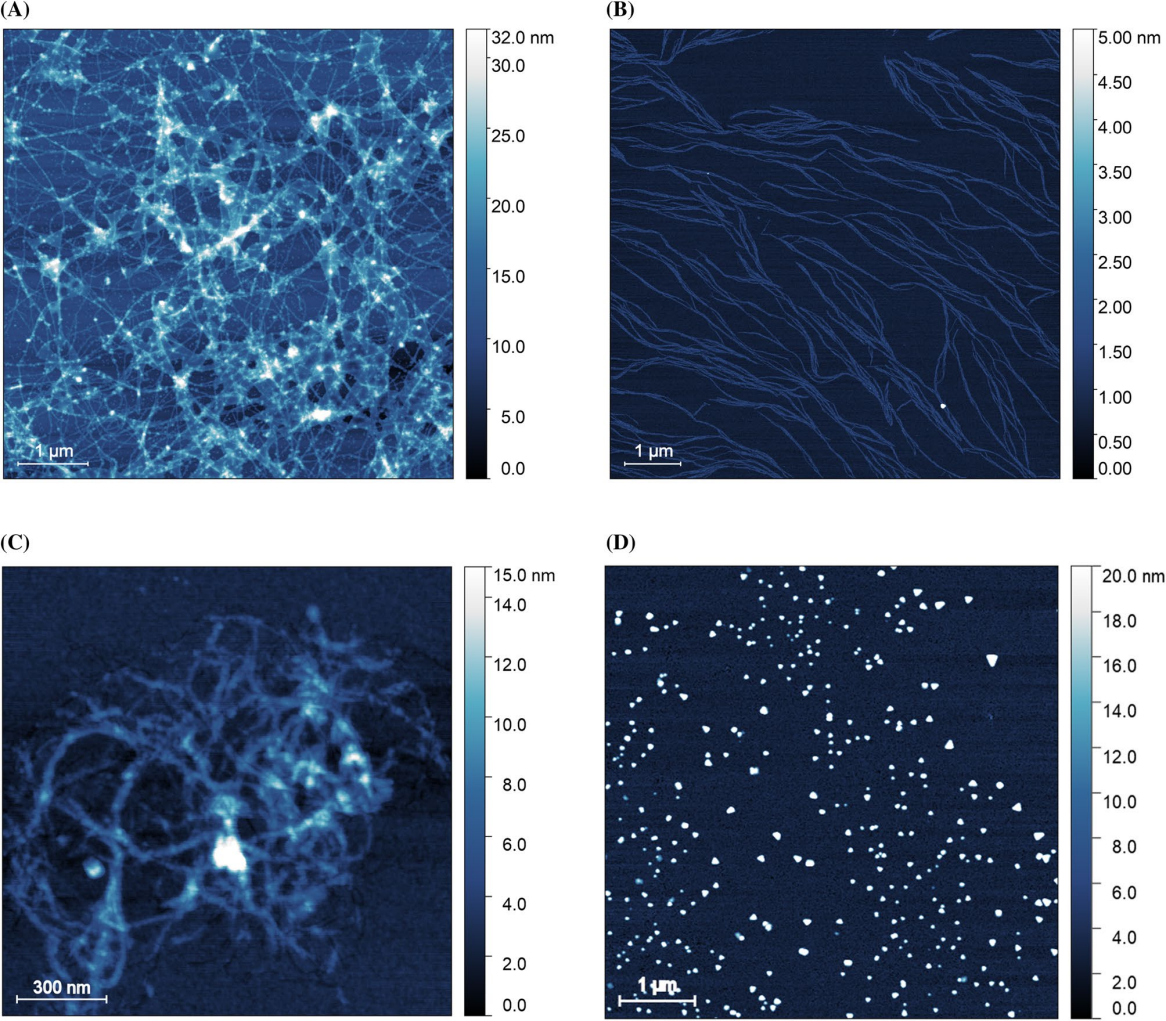
AFM images of Aβ alone and Aβ after incubation with HCC samples. (**A**) Aβ 1–42 fibrils; (**B**) Aβ 3–28 fibrils; (**C**) Aβ 1–42 after overnight incubation with HCC; (**D**) Aβ 3–28 after overnight incubation with HCC.

The observations from the SANS experiments, exhibiting the direct participation of human cystatin C molecules in the disaggregation process of selected Aβ peptides, were verified using atomic force microscopy. For this purpose, samples identical to those used in the SANS experiments were prepared for both the fibrillated peptides (Aβ 3–28 and Aβ 1–42) alone as well as in mixtures with human cystatin C. Samples were then incubated for approximately 9 h and further investigated by AFM. The incubation time was chosen to correlate with the full time of the SANS kinetic experiment. The representative results of AFM studies are presented in Fig. 7 and Figs. S3, S4, S5 and S6 from Supplementary materials. Both Aβ peptides form distinct fibrillar structures. The Aβ 1–42 fibrils (Fig. 7A) have an average height of up to 3 nm and are entangled and assembled into irregular bundles. The Aβ 3–28 peptide forms uniform fibrils with a maximum height of up to approximately 2.5–3 nm and several micrometers in length (see Fig. 7B). Such fibrillar structures have been previously observed for Aβ 1–28^75,76^ and Aβ 1–42^63^ peptides.

The aim of these experiments was to track and visualize the disaggregation process of the Aβ 3–28 and Aβ 1–42 peptide fibrils in the presence of HCC. The data obtained by AFM topography studies of aggregates formed after overnight incubation of both types of fibrils with human cystatin C are shown in Fig. 7C,D. In the case of Aβ 1–42 peptide fibrils, incubation in the presence of HCC resulted in only minor morphological changes. The fibrillar structure was preserved, and an analysis of the AFM images shows that human cystatin C molecules probably adhere to Aβ 1–42 peptide fibrils. We base this assumption on the presence of HCC in amyloid β deposits observed by Levy et al. ^19^. However, based solely on the results of AFM and SANS studies, we cannot conclusively confirm the direct deposition of HCC on Aβ 1–42 fibrils.

The most important observation from AFM imaging is that the fibrils of the Aβ 3–28 variant appear to be disaggregated after incubation with human cystatin C. The characteristic morphology (uniform fibrils observed for Aβ 3–28 alone) disappears completely (see Fig. 7D). They are replaced by spheroid-like aggregates of height up to 20 nm and diameter 50 to 200 nm. This result agrees well with the earlier observations from the SANS kinetic studies.

## Discussion

Aβ peptides are the major component of senile plaques and are the fingerprint of Alzheimer's disease^1^. Aβ fibrils are already widely characterized by a variety of methods, such as TEM^63^ NMR^77^, AFM^78^, and thioflavin assays^79^. However, structural studies of Aβ peptides and their binding to other molecules are difficult due to the demanding nature of the sample. The method of choice, SANS with the use of contrast match^80^, turns out to be a great experimental method for tracking structural changes in amyloid fibrils, including the interactions between fibrils and other biomolecules^72,81^. Combining the SANS methodology with other techniques, such as AFM, we are able to obtain a full picture of the possible interaction and impact of different biomolecules on the aggregation of amyloid-beta peptides.

The different Aβ peptides are produced in sequential cleavage by secretase enzymes in the process of APP degradation^81^. The senile plaques of the Aβ peptide are composed of Aβ 1–40, Aβ 1–38, Aβ 1–42, Aβ 1–37, and Aβ 1–43. The shorter variant 1–15/16 also appears due to the cleavage of APP; however, it does not take part in forming aggregates^82^. Aβ 1–28 was characterized as a predominantly helical part of the transmembrane domain^83^.

The shorter Aβ 3–28 variant sequence (with the shorter hydrophobic C-terminal end) causes the fibrils to be less hydrophobic, less condensed, more exposed to interactions, and more sensitive to the structural transition. The length of Aβ peptides and conformational state can determine neurotoxicity, aggregation tendency or interactions with other biomolecules^84^. The results obtained in this study indicate that, during interaction with amyloid fibrils, HCC not only inhibits aggregation^85^. This is one of the probable reasons why HCC was discovered as a codeposit of senile plaques^19^. In addition, HCC can penetrate and depolymerize fibrils of the Aβ 3–28 variant, which means that the aggregation can be reversed. We suspect that HCC, as a part of senile plaques, may take part in the disaggregation of fibrils or old aggregates. However, it is worth referring here to the studies conducted by Tizon et al., which showed that the extracellular addition of HCC and preformed Aβ oligomers or fibrils increased cell survival. HCC inhibits Aβ aggregation, unfortunately it is not able to dissolve formed Aβ fibrils or oligomers^44^.

**Figure 8:**
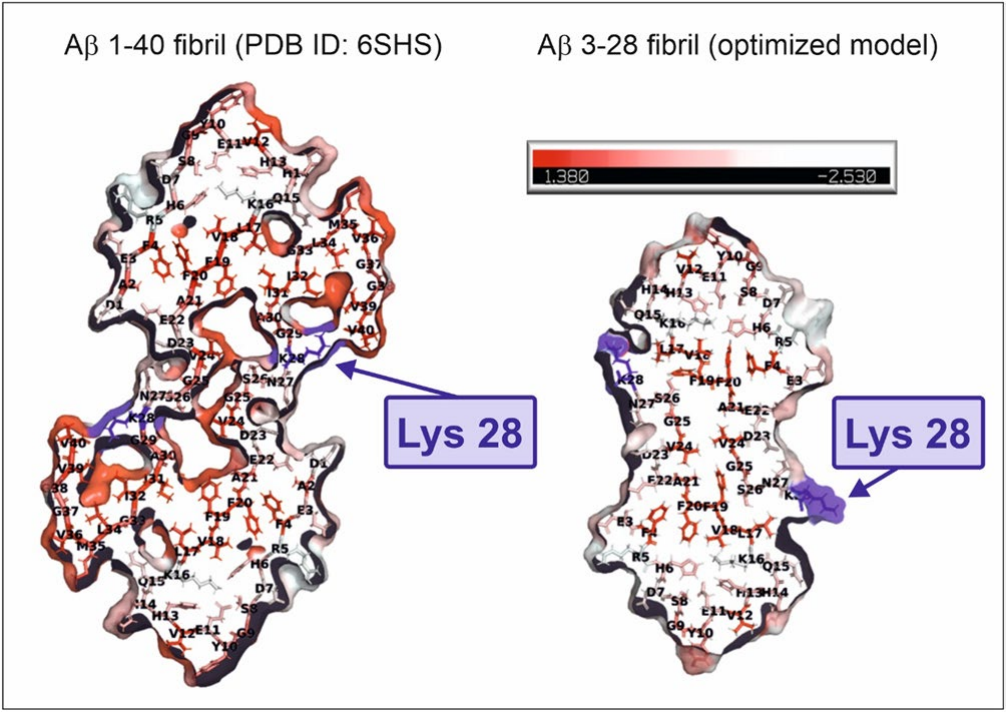
The structures of 1st layer of fibrils coloured according to hydrophobicity (red) of amino acids^89^ with lysine 28 marked (blue). In a model of Aβ 3–28, Lys28 is exposed to solution and is not stabilized by interactions with other amino acids. The figure was created using PyMOL Molecular Graphics System, (Schrödinger, LLC).

A number of published studies describe the Lys28 amino acid as crucial for amyloid fibril formation due to the Lys28-Asp23 and/or Lys28-Glu22 salt bridges^47,86^. The intramolecular interaction between these amino acids driven by a combination of hydrophobic and electrostatic interactions is important. It was shown that Zn^2+^ ions can break an Asp23-Lys28 salt bridge^87^. In the modelled fibril structure, the Glu22 and Asp23 amino acids can interact by electrostatic interactions when Lys28 stabilizes the loop conformations. Moreover, fibril formation is probably driven by the turn motif of Gly25, Ser26, Asn27, the hydrophobic interaction of Val24, and the side chain of Lys28. In the presented fibril structure Aβ 1–42, Lys28 stabilizes the loop together with Val40 by hydrophobic interaction; however, in the case of variant Aβ 3–28, the C-end is missing, and Lys28 is exposed. Fibril depolymerization of variant 3–28 observed during our experiments can be associated with the fact that Lys28 is not involved in structure stabilization but is exposed to electrostatic and hydrophobic interactions with HCC (Fig. 8).

Analysing the model of the Aβ 3–28 fibril, we can see that its core is stabilized by hydrophobic amino acids. HCC molecules can also, under certain conditions, undergo conformational changes to expose a more hydrophobic interface. The structure of HCC in its native monomeric form is held together by hydrophobic forces^88^. At this point, it is worth considering how the HCC molecule can break down the structure of Aβ fibrils. Human cystatin C molecules are flexible and undergo conformational changes through the mechanism of domain swapping. The fragment of the HCC molecule located in the region from Ile56 to Gly59, which corresponds to hinge loop L1 in the sequence of the human cystatin monomer^23^, is responsible for the flexibility of this structure. However, even the native HCC dimer structures in the polymorphic crystals show differences, forming a dimer with an open conformation (PDB code: 1TIJ) and a compact conformation (PDB code: 1G96). Therefore, it should be assumed that the abovementioned flexibility of the HCC conformation allows the exposure of HCC hydrophobic areas that can interact with the surface of the Aβ peptide fibrils. On the other hand, the N-terminal fragment of HCC is highly flexible and could also be involved in the penetration and disruption of the Aβ fibril structure. The structure of such a flexible, unstructured N-terminal HCC fragment was proposed by Perlenfein et al.^33^. The phenomenon of disaggregation of 3–28 Aβ fibrils by HCC may be driven by disruptions of the hydrophobic forces and dynamic codestabilization between those molecules. Therefore, it can be assumed that the flexible N-terminal fragment of the HCC structure may interact with some amyloid beta fibrils, inducing their disaggregation. Additionally, both of the described effects can probably act synergistically.

Further investigation of the disaggregation properties of HCC towards amyloid fibrils formed by other variants of Aβ peptide can provide more answers about the molecular mechanisms underlying the protective properties of HCC. Of extreme interest would be to answer some newly arisen questions: (i) whether the process of disaggregation of the amyloid fibrils by HCC occurs naturally and whether it depends on the level of expression of HCC in CSF^17^, (ii) which of the known, naturally occurring sequences of Aβ peptides (besides Aβ 1–42) are resistant to disaggregation by HCC and whether this resistance can depend on different structures of the steric zipper formed by those peptides.

The process of disaggregating amyloid beta peptide fibres can be induced by the presence of various molecules, ranging from small molecules such as surfactants to large protein molecules^90–94^. Recently several small molecules, designed using a joint pharmacophore space (JPS) method, were successfully used in the disassembling of Aβ oligomers and protofibrils^95^. The observed in this study changes were similar to our AFM imaging results, showing the disappearance of Aβ 3–28 fibrils induced by HCC. Particularly the gradual disaggregation of Aβ fibril structures by the drug candidates (eg. AC0201) reported by Jin et al.^95^ and formation of oligomers or aggregates was visible there. Many biomolecules have been investigated in clinical trials of Aβ peptide targeting therapy and the reduction of amyloid deposits through degradation and removal. There are also studies aimed at inducing such processes as the inhibition of protein aggregation through the participation of antibodies and enzyme inhibitors^96,97^. To date, scientists have designed several inhibitors of Aβ and Tau aggregation, including the promising anti-Aβ monoclonal antibody aducanumab, which is now a treatment endorsed by the Food and Drug Administration (FDA)^98^. We believe that antibodies or peptides inspired by proteins such as HCC may be of therapeutic importance for the treatment and elimination of amyloidogenic peptides or the digestion of already present senile plaques. Designing new disaggregation strategies is crucial because they are effective in combating and possibly causing regression of the disease when the brain of the individual is in an advanced pathological state.

Summing up the results of our research, we would like to emphasize that they are part of the current trend in the characterization of HCC interactions with Aβ peptides. The growing interest in cystatin as an Aβ binding agent dates back to the early twenty-first century when Levy and co-workers identified it in deposits with amyloid-beta protein in the brain of Alzheimer disease patients^75^. It is also worth noting that Kaeser et al. observed that cystatin C overexpression in brains of transgenic mice reduced the deposition of β-amyloid^42^. However, Chen et al.^99^ observed that elevated level of cystatin C in serum of AD patients correlated with the disease progress. Moreover Mi et al.^100^ reported the association of soluble forms of HCC with Aβ 1–42. Therefore, the continuation of further work leading to a full understanding of the impact of HCC on the amyloidogenic processes is necessary.

Therefore, we hope that our observations on the effect of HCC on the disaggregation of the Aβ 3–28 peptide will become an inspiration for the search for new methods of therapy for Alzheimer's disease.

### Supplementary Information

Supplementary Figures.

## Data Availability

The datasets used and analysed during the current study available from the corresponding author on reasonable request.
