# Supplementary material for: Human cystatin C induces the disaggregation process of selected amyloid beta peptides: a structural and kinetic view
Source: Sci Rep. 2023 Nov 27;13:20833. doi: 10.1038/s41598-023-47514-w (PMC10682421; doi:10.1038/s41598-023-47514-w)
Supplement: Supplementary file 1 — Supplementary Figures. [file 41598_2023_47514_MOESM1_ESM.pdf]

Supporting information for:

HUMAN CYSTATIN C INDUCES DISAGREGATION PROCESS OF SELECTED AMYLOID BETA PEPTIDES – A STRUCTURAL AND KINETIC VIEW

Adriana Żyła[a,b], Anne Martel[c], Przemysław Jurczak[d], Augustyn Moliński[a,b], Aneta Szymańska[d], Maciej Kozak[a,e*]

[a)] Department of Biomedical Physics, Faculty of Physics, Adam Mickiewicz University, Poznan, Poland

[b)] NanoBioMedical Centre, Adam Mickiewicz University, Poznan, Poland

[c)] Large Scale Structures, ILL Neutrons for Society, Institute Laue-Langevin, Grenoble, France

[d)] Laboratory of Medical Chemistry, Department of Biomedical Chemistry, Faculty of Chemistry, University of Gdansk, Poland

A)

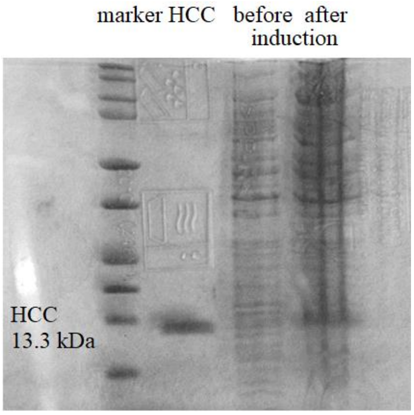

B)

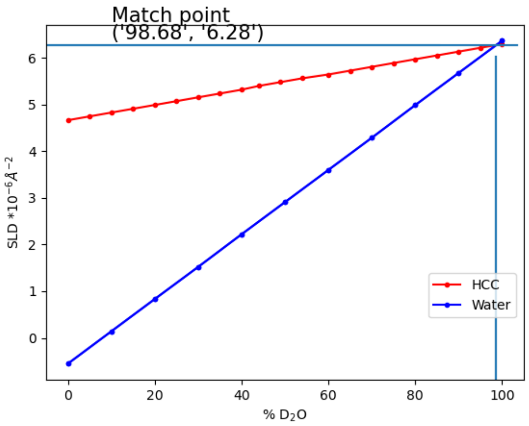

C)

sample	Observed mass (Da)	Expected mass (Da)	Mass deviation (Da)	Mass accuracy (ppm)	interpretation
D-Human Cystatin C	13898 Da	H: 13347.18; D-protein in H2O 14044.53	-	-	Using the following formula %D (H20)=100*(MexpH-MHH)/(M DH-MHH), the sample is deuterated at 79%

D)

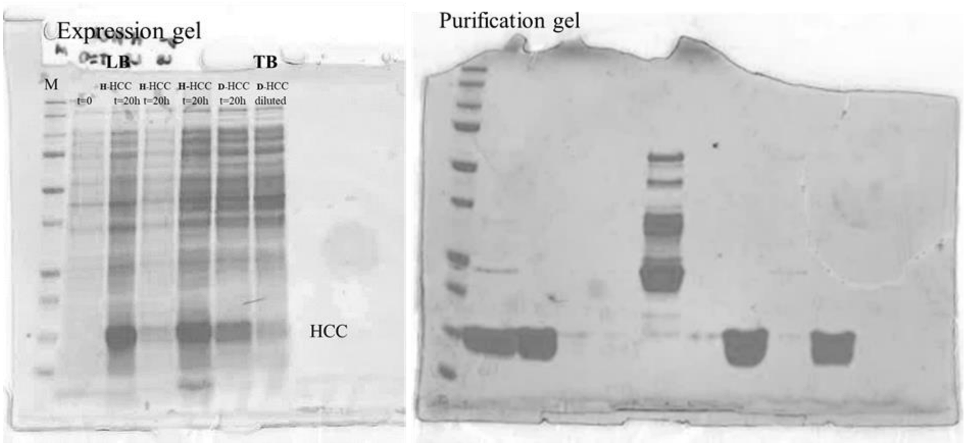

,

Figure S1. Overexpression of deuterium-labeled Human Cystatin C in LB. A) Overnight growth of bacteria at D_2O media: SDS page analysis of bacteria protein composition before and after induction. As a control pure HCC B) Theoretical contrast match for 79% Deuterated Human Cystatin C (D-HCC) (Biomolecular Scattering Length Density Calculator) C) Mass spectroscopy report indicating the level of deuteration produced D-HCC. D) Original gels from expression and purification.

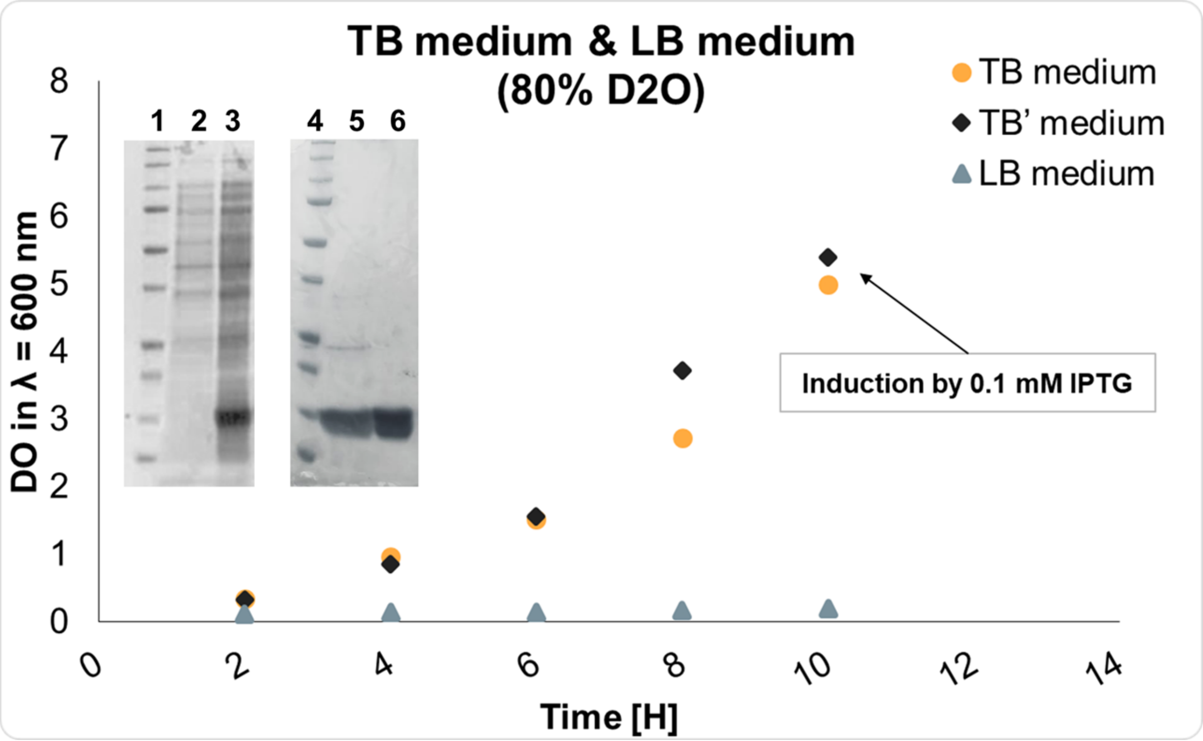

Figure S2. The plot shows a Shuffle T7B cell grows at LB media and TB media. The cells grow much faster in TB medium than in LB. The DO 5 was reached after around 10 hours of growth time at 30 °C. The SDS page analysis: 1) Marker 2) fraction before induction 3) fraction after IPTG induction 4) Fraction after IEX chromatography 5) Pure protein after SEC chromatography

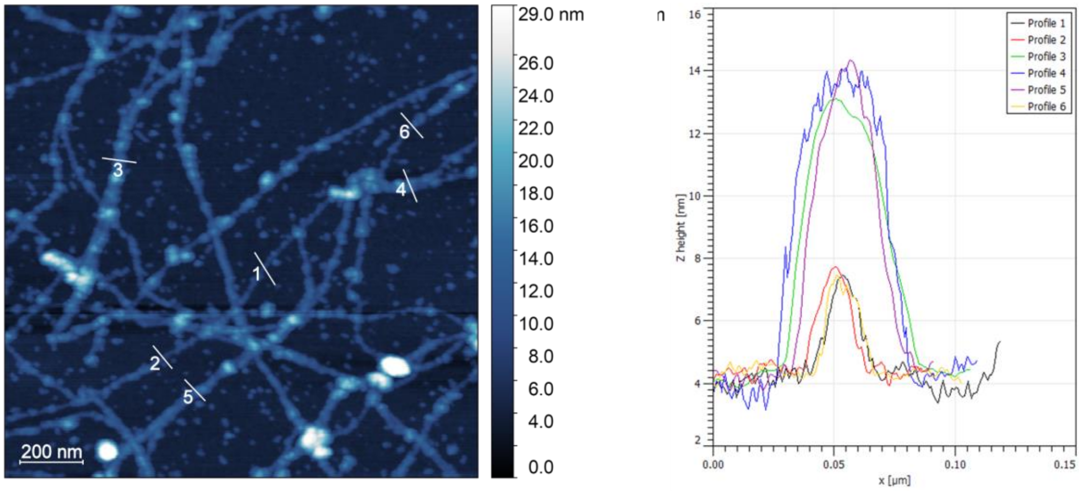

Figure S3. AFM profile of fibrils Aβ 1-42.

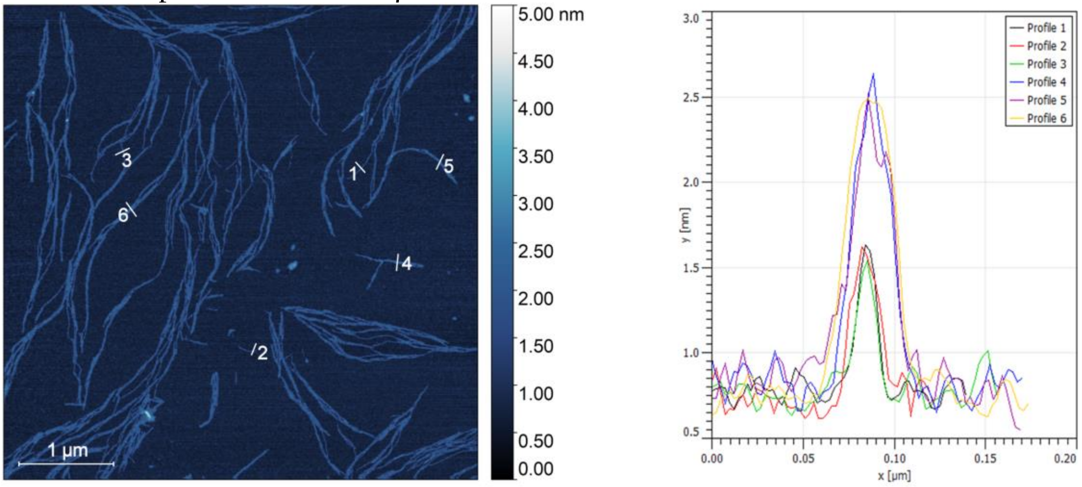

Figure S4. AFM profile of fibrils Aβ 3-28.

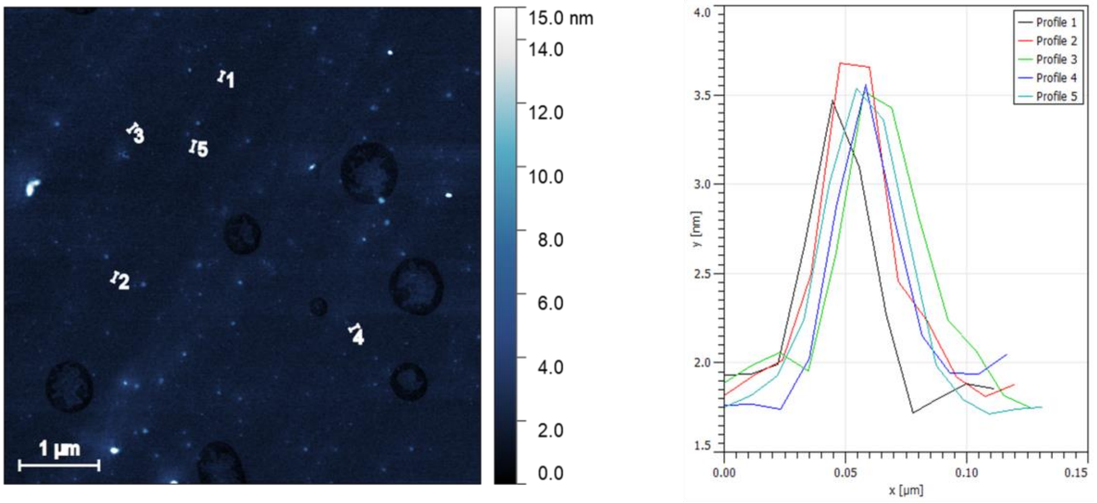

Figure S5. AFM profile of HCC alone.

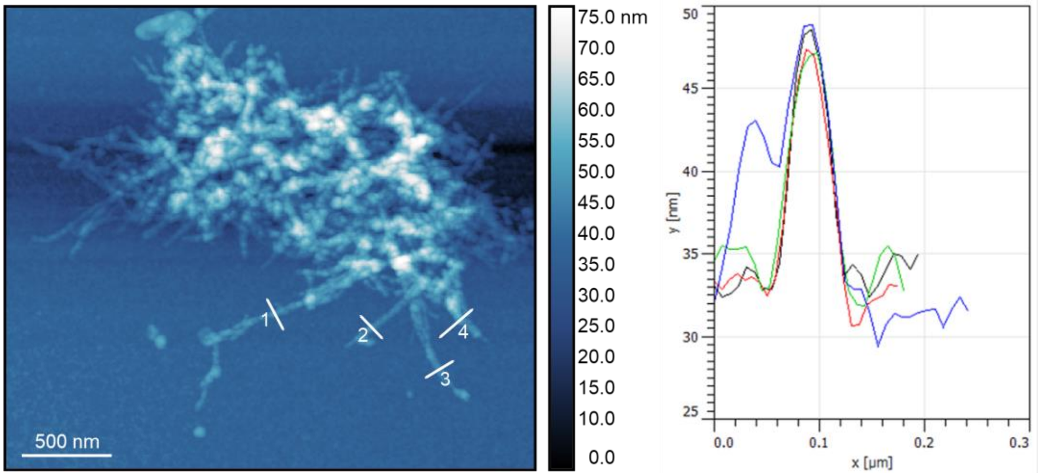

Figure S6. AFM image of Aβ1-42 fibrils in the presence of cystatin and selected profiles.

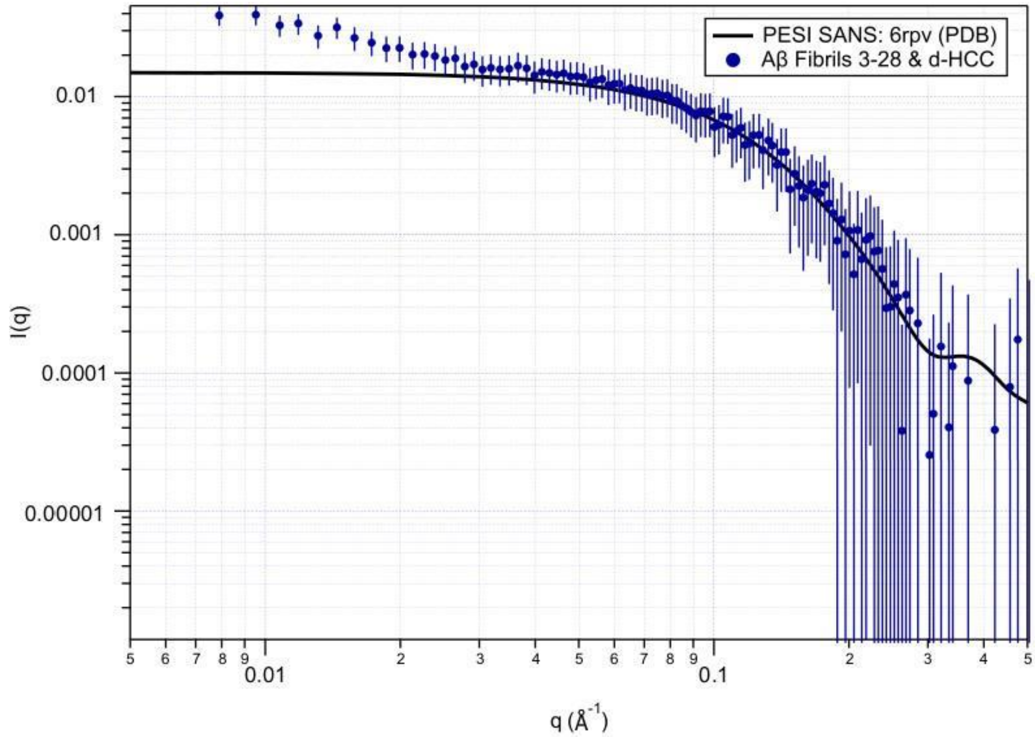

Figure S7. The small-angle neutron scattering curve of 3-28 fibrils (matched out) and D-HCC at 45% D_2O (contrast point for proteins).

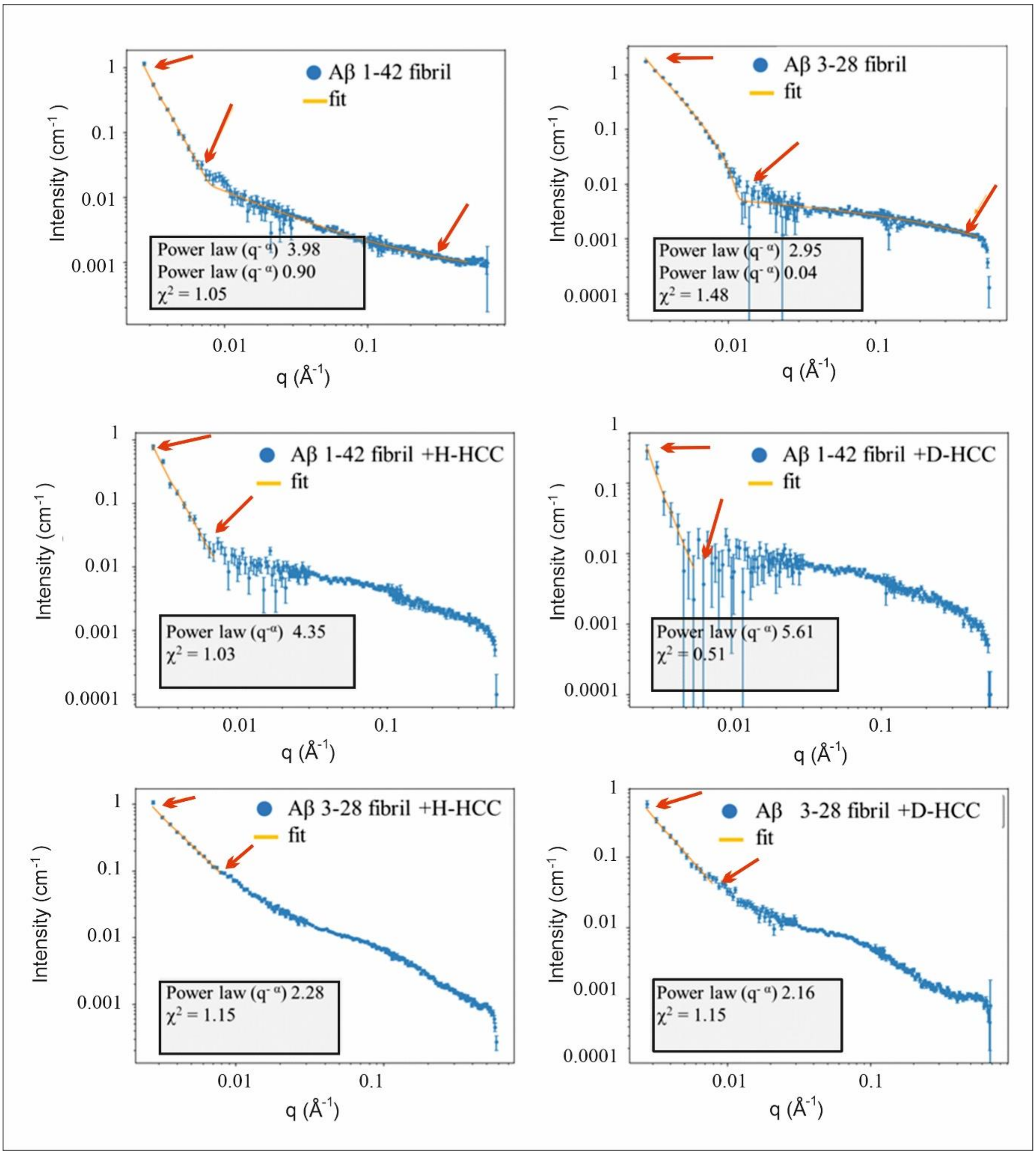

Figure S8. Fitting a slope with power law for small-angle neutron scattering (SANS) experiment curves from amyloid fibrils in presence of H-HCC and D-HCC up: Aβ 1-42; down: Aβ 3-28 fibrils

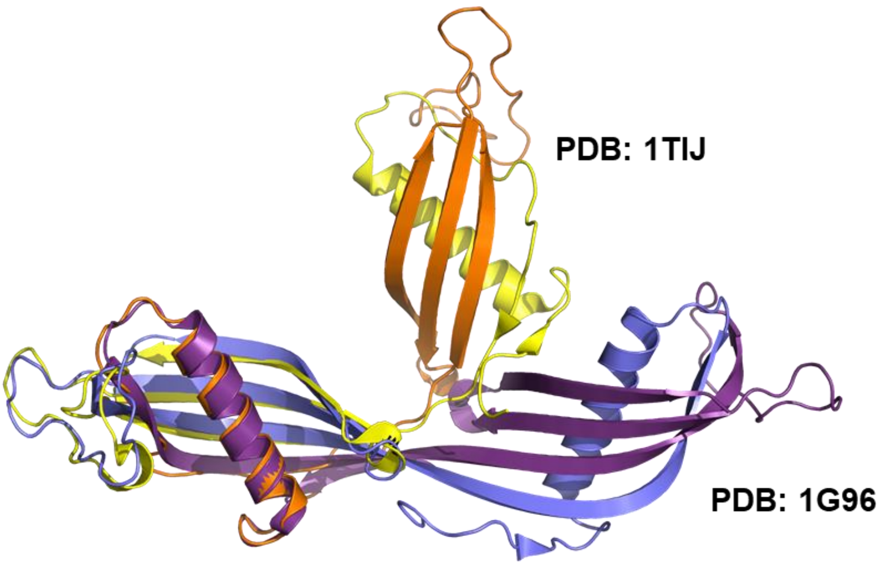

Figure S9. HCC domain swapping structures: superposition of two dimeric structures of HCC. 1TIJ: yellow – chain A, orange – chain B; 1G96: violet – chain A, blue – chain B
